# HSP90 is essential for Jak-STAT signaling in classical Hodgkin lymphoma cells

**DOI:** 10.1186/1478-811X-7-17

**Published:** 2009-07-16

**Authors:** Nils Schoof, Frederike von Bonin, Lorenz Trümper, Dieter Kube

**Affiliations:** 1 Department of Hematology and Oncology, Medical Center of the Georg-August-University Göttingen, Göttingen, Germany

## Abstract

In classical Hodgkin lymphoma (cHL) chemotherapeutic regimens are associated with stagnant rates of secondary malignancies requiring the development of new therapeutic strategies. We and others have shown that permanently activated Signal Transducer and Activator of Transcription (STAT) molecules are essential for cHL cells. Recently an overexpression of heat-shock protein 90 (HSP90) in cHL cells has been shown and inhibition of HSP90 seems to affect cHL cell survival. Here we analysed the effects of HSP90 inhibition by geldanamycin derivative 17-AAG or RNA interference (RNAi) on aberrant Jak-STAT signaling in cHL cells. Treatment of cHL cell lines with 17-AAG led to reduced cell proliferation and a complete inhibition of STAT1, -3, -5 and -6 tyrosine phosphorylation probably as a result of reduced protein expression of Janus kinases (Jaks). RNAi-mediated inhibition of HSP90 showed similar effects on Jak-STAT signaling in L428 cHL cells. These results suggest a central role of HSP90 in permanently activated Jak-STAT signaling in cHL cells. Therapeutics targeting HSP90 may be a promising strategy in cHL and other cancer entities associated with deregulated Jak-STAT pathway activation.

## Findings

**Figure 1:**
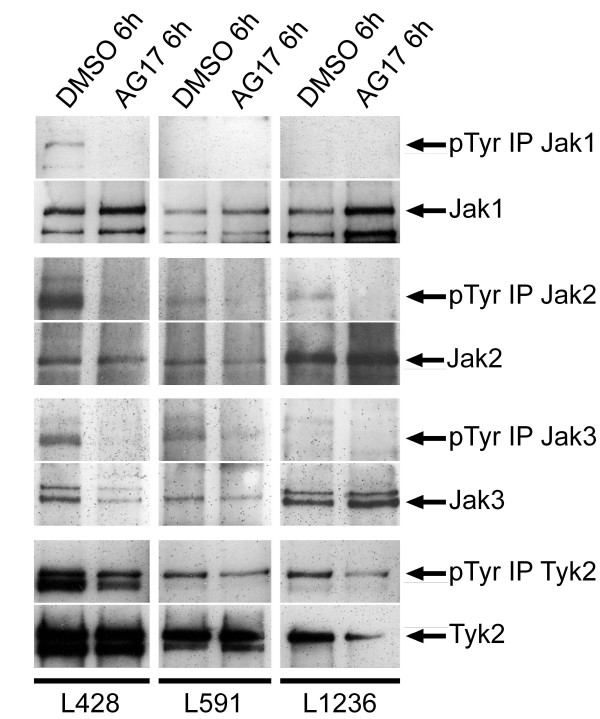
**Tyrphostine AG17 leads to dephosphorylation of Janus kinases after 6 h of treatment in cHL cell lines**. Cell lines L428, L591 and L1236 were incubated with DMSO or 10 μM AG17 for 6 h as described recently [2]. Tyrosine phosphorylation of Jaks was analysed by immunoprecipitation (Protein A Microbeads, Miltenyi Biotech) with Jak specific antibodys (Jak1 (HR-785), Jak2 (C-20), Jak3 (C-21) and Tyk2 (C-20) Santa-Cruz) and immunostaining of tyrosine phosphorylation by 4G10 antibody (Upstate). Loading controls were accomplished by specific antibodys against Jak proteins.

Classical Hodgkin lymphoma (cHL) is one of the most frequent lymphoid tumours predominantly derived from germinal centre B cells. Despite high survival rates, observations of long-term survivors reveal a high incidence of chemotherapy-related secondary malignancies as well as a stagnant rate of relapses and non-responders. Permanent activation of Signal Transducer and Activator of Transcription (STAT) molecules is essential in a number of malignancies regulating cell proliferation, apoptosis, immune escape and tumor angiogenesis. Especially STAT3 and STAT6 but probably also STAT5 seem to be associated with cell proliferation in cHL cell lines of B and T cell origin [1,2]. The main activators of STAT molecules are cytokine receptor associated Janus kinases (Jaks). Cochet and co-workers could show a permanent activation of Jak1, 2 or 3 in two T cell derived cHL cell lines involved in cHL cell survival [3]. As shown in Figure 1, a comparable activation of Jaks could be observed in B-cell derived cHL cell lines. Especially Jak2 and Tyk2 are found in a phosphorylated state in L428, L591 and L1236 cells. Incubation with tyrphostin AG17, which is capable in inhibition of STAT phosphorylation in cHL cells [2], is associated with a loss of phosphorylation in Jak1, Jak2 and Jak3 as well as a reduction of phosphorylation in Tyk2 within 6 h. However, it is possible that reduction of Jak phosphorylation is also partially due to a reduction of Jak protein levels upon AG17 treatment but the reduction of phosphorylation is much stronger compared to reduction of Jak protein levels.

Recently, HSP90-inhibitors geldanamycin and its derivative 17-AAG have been shown to have anti-proliferative effects, sensitize for apoptotic stimuli and synergize with chemotherapy regimens in cHL cell lines [4,5]. It was suggested that HSP90 inhibitors could be used for treatment of relapsed lymphoma patients in order to substantially reduce the toxic burden of standard therapies. HSP90 is a chaperone essential for maturation and stabilization of multiple client proteins. Thereby it is associated with crucial cellular functions like cell proliferation and survival by chaperoning key proteins of respective signaling pathways. The frequently observed overexpression of HSP90 in certain neoplasias probably leads to a stabilisation of proto-oncogenes or sustained aberrant signaling in the malignant cells. Jak1 and Jak2 have been described to interact with HSP90 and could therefore be involved in a permanent Jak-STAT signaling in cHL [6]. Due to the overexpression of HSP90 [4] and the permanent activation of Jak-STAT signaling [1-3,7,8], we asked the question if HSP90 supports permanent Jak-STAT signaling in cHL cells. Inhibition of HSP90 could be associated with the interruption of permanent STAT activation supporting the idea to use HSP90 inhibitors in multi-targeting tumor therapies. Therefore, we analysed the effects of the HSP90-inhibitor 17-AAG as well as RNA-interference mediated inhibition of HSP90 on the tyrosine phosphorylation of STAT1, STAT3, STAT5, STAT6 and protein expression of Jak1, Jak2, Jak3 and Tyk2 in cHL cells.

**Figure 2:**
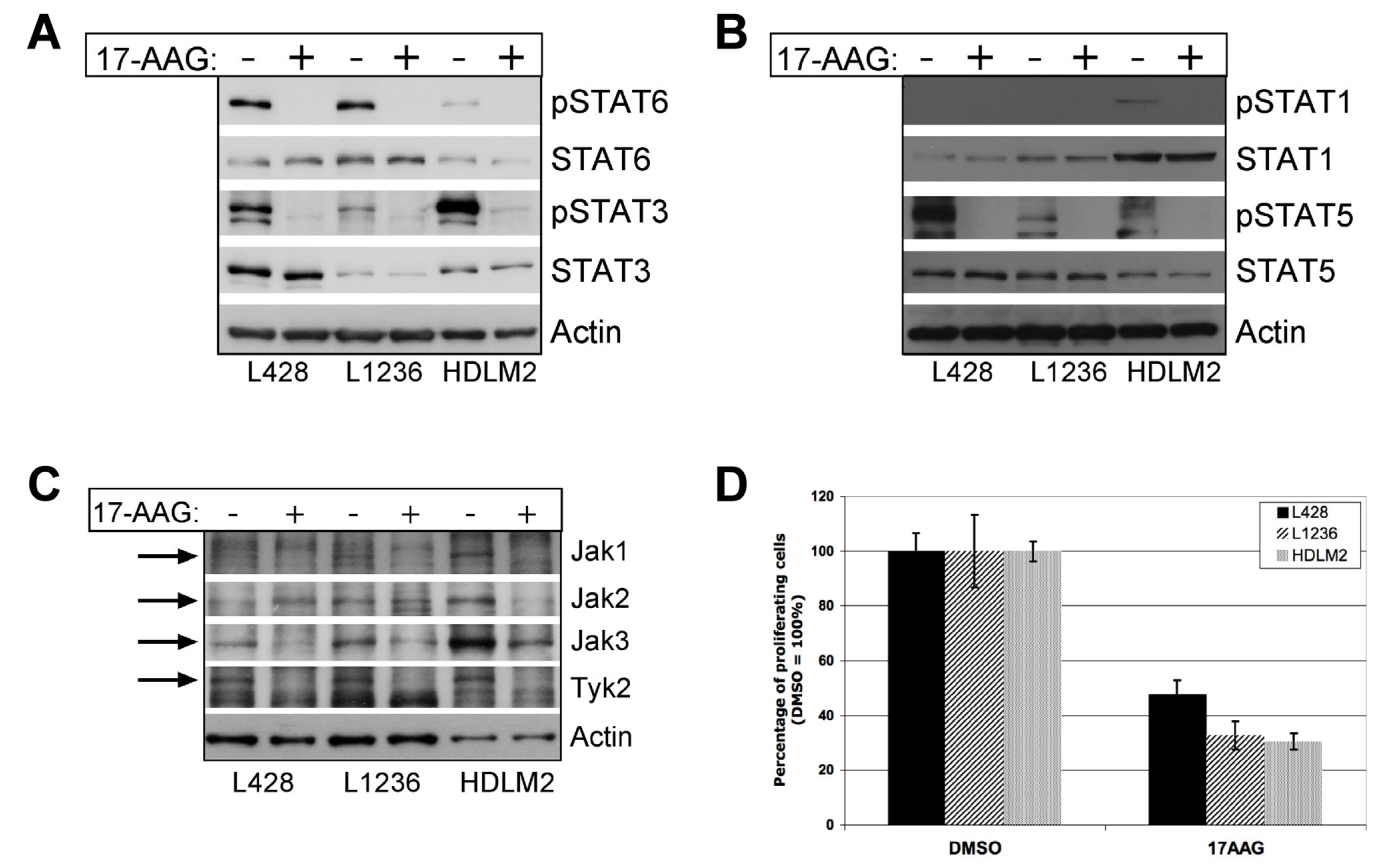
**17-AAG inhibits phosphorylation of STAT1, STAT3, STAT5 and STAT6 (A + B), reduces expression of Jak1, Jak3 and Tyk2 (C) and cHL cell proliferation (D)**. cHL cell lines L428, L1236 and HDLM2 were treated with DMSO (Sigma-Aldrich) or 5 μM 17-AAG (17-(Allylamino)-17-demethoxygeldanamycin, Merck/Calbiochem) for 24 h. STAT tyrosine phosphorylation and Jak expression was analysed by immunoblot as described recently [2]. (A + B) Phosphorylation of STAT3 (Tyr705) (#9131, Cell Signaling Technology), STAT6 (Tyr641) (#9366, Cell Signaling Technology) (A), STAT1 (Tyr701) (#9172, Cell Signaling Technology) and STAT5 (Tyr694) (#9351, Cell Signaling Technology) (B) is completely abrogated within 24 h. STAT1 tyrosine phosphorylation was only detectable in HDLM2 cells. Protein levels of the STAT molcules remain mostly unaltered (Antibodies: STAT1 (#9171) STAT3 (#9132) (Cell Signaling Technology); STAT5 (610191), STAT6 (610291) (BD Transduction Laboratories)) (C) 17-AAG leads to strong reduction of Jak1, Jak3 and Tyk2 expression in L428, L1236 and HDLM2 cells. Jak2 is enhanced in L428 and L1236 cells and reduced in HDLM2 cells. (D) Incubation of cHL cells with 17-AAG leads strong inhibition of cHL cell proliferation. cHL cell proliferation was measured by ^3^H-Thymidin incorporation after 24 h of 17-AAG treatment. ^3^H-Thymidin was added 16 h – 18 h before harvesting. The data is depicted as percentages (DMSO = 100%) and error bars mark the standard deviation of three replications.

First cHL cell lines L428, L1236 and HDLM2 were incubated with 5 μM 17-AAG or DMSO. Cell proliferation was measured by 3H-Thymidin incorporation and cells were harvested after 24 h of 17-AAG treatment. Whole cell extracts were prepared and analysed by immunoblot. As shown in Figure 2A, incubation of cHL cell lines L428, L1236 and HDLM2 with 17-AAG is accompanied by an entire loss of STAT3 and STAT6 tyrosine phosphorylation in all three cHL cell lines compared to DMSO treated cells. STAT1 tyrosine phosphorylation (only detectable in HDLM2 cells) and STAT5 tyrosine phosphorylation were also abrogated after 24 h of 17-AAG treatment (Figure 2B). Expression of STAT seems to be unaffected by 17-AAG treatment as shown by unaltered protein amounts (Figure 2A &2B). Due to the potential chaperone functions of HSP90, we therefore examined the effects of 17-AAG on protein amount of Jak1, Jak2, Jak3 and Tyk2 in cHL cells compared to DMSO treated cells (Figure 2C). Within 24 h of 17-AAG treatment the amount of detectable Jak1, Jak3 and Tyk2 protein was reduced in all three analysed cHL cell lines. Expression of Jak2 was slightly enhanced in cHL cell line L428 and L1236 not affecting inhibition of permanent tyrosine phosphorylated STATs, but abrogated in HDLM2 cells. In Figure 2D the strong inhibition of cell proliferation is shown, which is in line with the literature [4,5].

**Figure 3:**
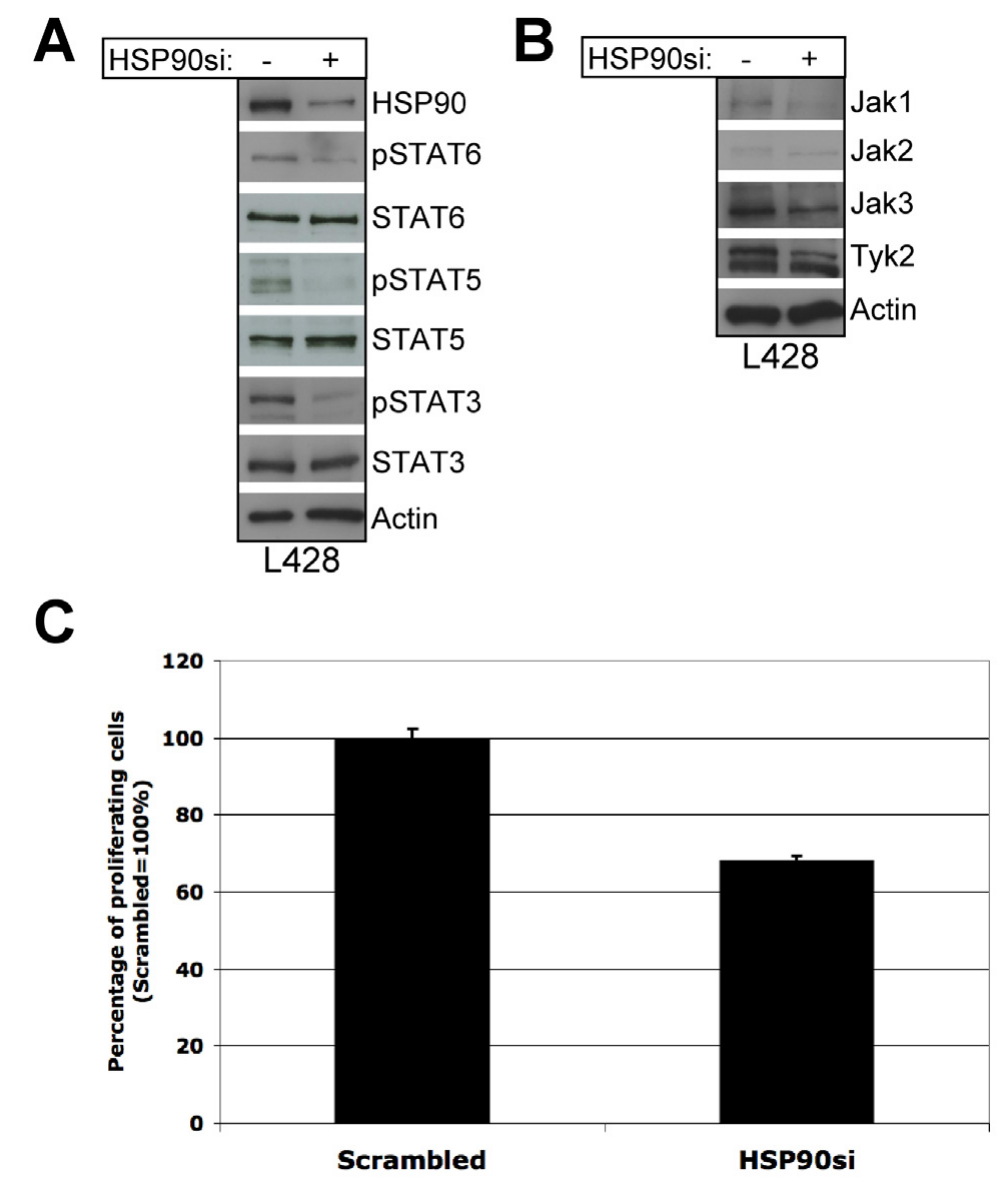
**Knock-down of HSP90 leads to reduced phosphorylation of STATs, downregulation of Jaks and reduced proliferation in L428 cells**. cHL cell line L428 was transfected with 3 μg siRNA against HSP90a& HSP90b or non-targeting scrambled siRNA (On-Targetplus, Dharmacon) by Nucleofection (Amaxa). Due to high expression levels of HSP90 nucleofection was repeated after 48 h. Cells were harvested 48 h after second nucleofection. Tyrosine phosphorylation of STAT3, -5 and -6 as well as protein expression of HSP90 (rat-anti-HSP90 mAB, Stressgen), STAT3, -5, -6, Jak1, -2, -3, Tyk2 and Actin was analysed by immunoblot. (A) Downregulation of HSP90 by siRNA reduced tyrosine phosphorylation of STAT3, -5 and -6 in L428 cells. (B) Downregulation of HSP90 by siRNA led to reduced protein expression of Jak1, -2, -3 and Tyk2 in L428 cells. (C) Reduced cell proliferation was observed by downregulation of HSP90 in L428 cells. cHL cell proliferation was measured by ^3^H-Thymidin incorporation after 48 h after second nucleofection. ^3^H-Thymidin was added 16 h – 18 h before harvesting. The data is depicted as percentages (Scrambled = 100%) and error bars mark the standard deviation of three replications.

In parallel both isoforms of HSP90 (HSP90a and HSP90b) were targeted by RNA-interference. Due to the strong expression levels of HSP90 in L428 cells siRNA transfection was performed twice by nucleofection. The transient knock-down of HSP90 in L428 cells confirmed the results obtained with 17-AAG treatment of cHL cells. HSP90 knock-down also showed a reduction of STAT3, -5 and -6 tyrosine phosphorylation without affecting protein levels of STATs (Figure 3A). STAT1 phosphorylation was not congruently detectable in L428 cells. Further analysis of Jaks in L428 cells also revealed a reduced expression of Jak1, Jak2, Jak3 and Tyk2 upon HSP90 knock-down (Figure 3B). In comparison to the results of 17-AAG treated cells, the knock-down of HSP90 seems to affect all 4 Jaks whereas 17-AAG had divergent effects on Jak2. As shown in Figure 3C a reduction of cell proliferation of L428 cells was visible after knock-down of HSP90. This effect was not as strong as observed in response to 17-AAG, which could be explained by remaining HSP90 protein expression due to an incomplete knock-down or due to additional effects of 17-AAG on HSP90-independent pathways.

HSP90 has been associated with the stabilization of mutant forms of oncogenes including the Jak2 mutation V617F in hematologic diseases [9]. Due to the observed permanent tyrosine phosphorylation of Jak2 and Tyk2 in cHL cell lines we analysed cHL cell lines for the presence of activating mutations. Jak2 V617F and Tyk2 V678F as well as Jak2 gene variations at D620E and E627E were sequenced within the cDNA of cHL cells as described previously [10-12]. In cHL cells lines L428, L1236, L591, KMH2, HDLM2 and L540 Jak2 is transcribed from the allele characterised by V617, D620 and E627, therefore no V617F activating mutations or other changes could be observed in the transcripts. The absence of Jak2 activating mutation V617F has recently been published [13]. The homologous mutation of Jak V617F in Tyk2 named V678F has been found to activate Tyk2 in the same manner as Jak2 V617F [11]. In all analysed cHL cell lines (L428, L591, L1236 and HDLM2) no Tyk2 V678F mutation was detected. Moreover, no other mutations within the sequenced cDNA regions were detected, suggesting no stabilization of mutant forms of Jak2 and Tyk2 by HSP90. This observation is in line with recent reports of autocrine activation of Jak-STAT signaling in cHL cells [1,14].

In summary, this study shows that HSP90 is important for the permanent activation of STAT molecules in cHL cells. Moreover, we conclude that the reduced protein levels of the Jaks upon inhibition of HSP90 are a major reason for the decreased STAT phosphorylation after inhibition of HSP90 by 17-AAG or RNAi against HSP90. Therefore, not only Jak1 or Jak2 as proposed by Shang and colleagues but also Jak3 and Tyk2 might be stabilised by HSP90 [6]. As proposed by Cochet and co-workers the activation of Jaks correlates with STAT activation in cHL cells [3]. This is in line with our finding that reduced protein amounts of Jaks lead to a reduced tyrosine phosphorylation of STATs in cHL cells. Nevertheless, we can not rule out that other HSP90 client proteins affect the phosphorylation of the STAT molecules or the expression of the Janus kinases, but HSP90 inhibition is accompanied by a strong reduction of permanent Jak-STAT signaling shown to be essential for cHL cell proliferation. Therefore, it is likely that the reduced cell proliferation upon HSP90 inhibition is mainly mediated by abrogation of permanent STAT activation.

Constitutive activation of NF-κB is another hallmark of cHL and could promote the expression of IL-13 and therefore activation of STATs [15]. The aberrant HSP90 expression in cHL cells could also be mediated by NF-κB and represent an additional mechanism to support permanent activation of STATs [16]. Thereby NF-kB could contribute to the malignant phenotype of cHL cells due to the stabilization of key proteins of multiple signaling pathways by HSP90 upregulation. Georgakis and co-workers showed an inhibition of PI3K/Akt and MEK pathways by 17-AAG in cHL cells which have been shown to contribute to survival of cHL cells to some extend [4]. The data presented here in conjunction with the recent publications outlines HSP90 as a potent therapeutical target in cHL patients of advanced stages or relapse. The abrogation of STAT phosphorylation of by HSP90 inhibition may be also useful for other cancer entities with permanently activated STAT molecules. Orally administered HSP90 inhibitors may open new perspectives in the treatment of these lymphoma by targeting multiple pathways and therefore eliminating the danger of resistance development observed in single targeted therapies as with imatinib mesylat.

## Competing interests

The authors declare that they have no competing interests.

## Authors' contributions

NS has made substantial contributions to conception and design of this work, acquisition, analysis and interpretation of data and in drafting the manuscript. FvB helped in the acquisition of the data. LT was involved in drafting the manuscript and revising it critically for important intellectual content. DK contributed to conception and design, interpretation of data as well as drafting the manuscript.
